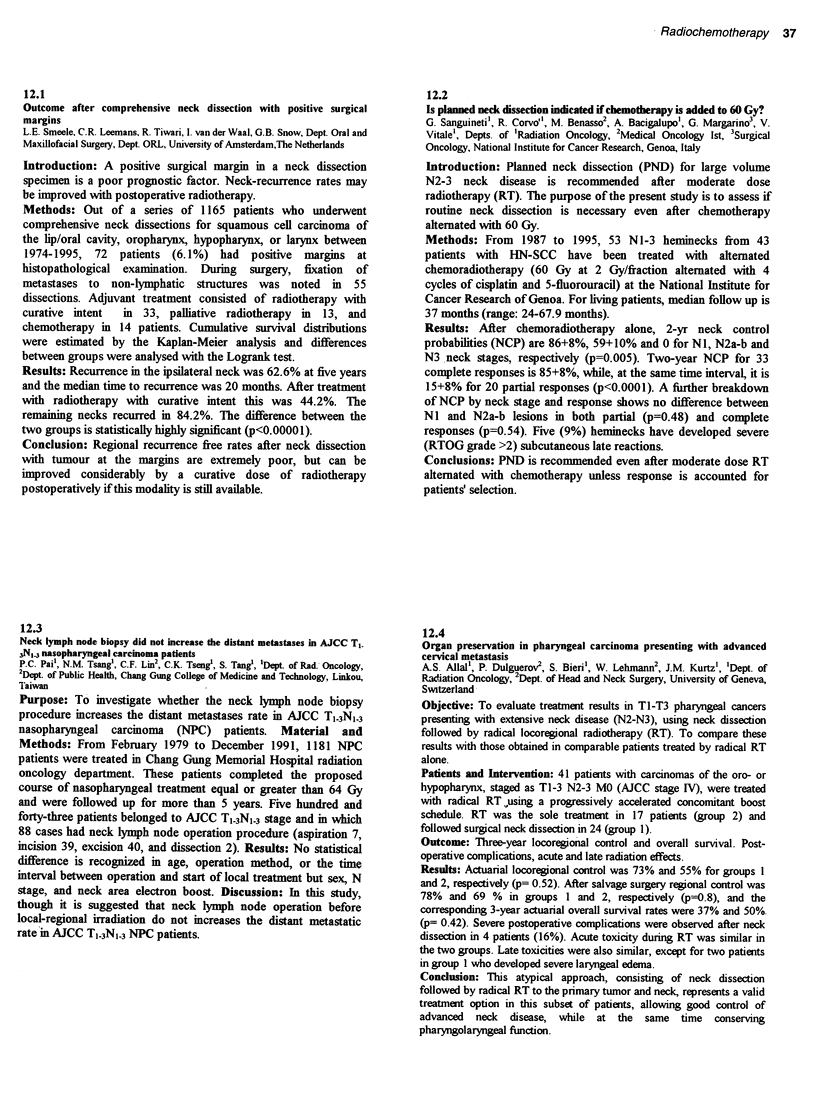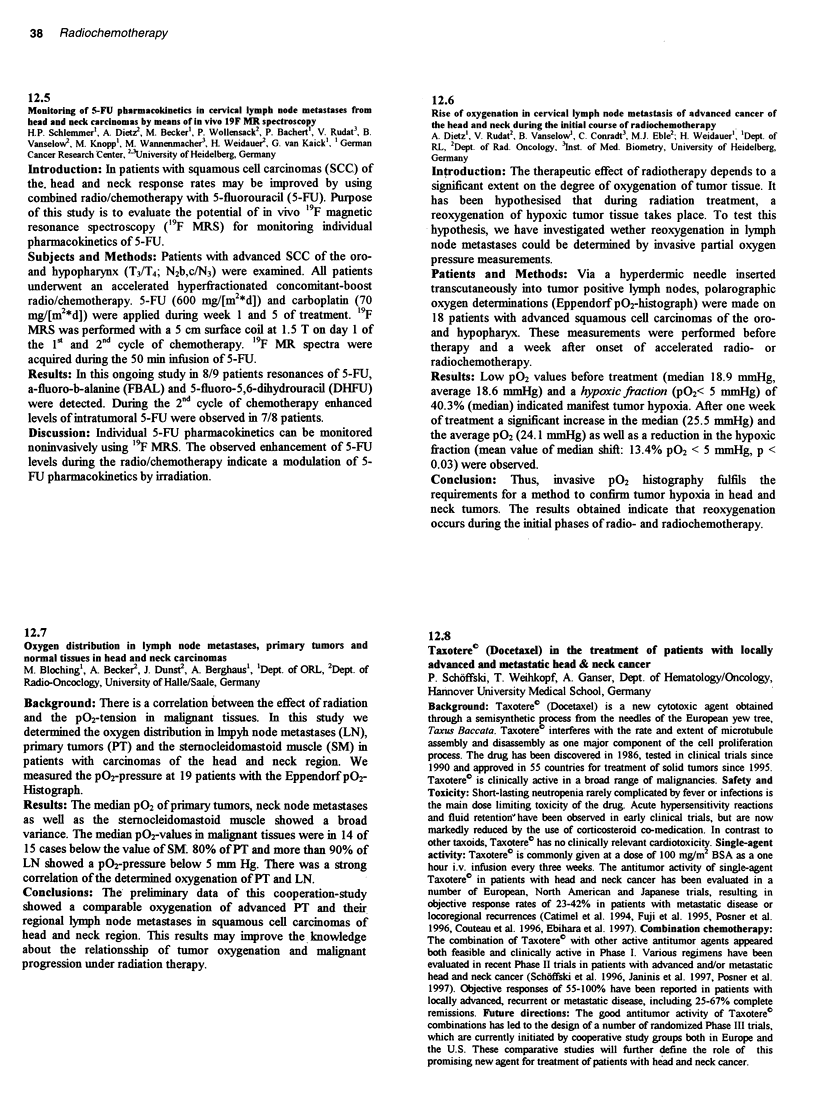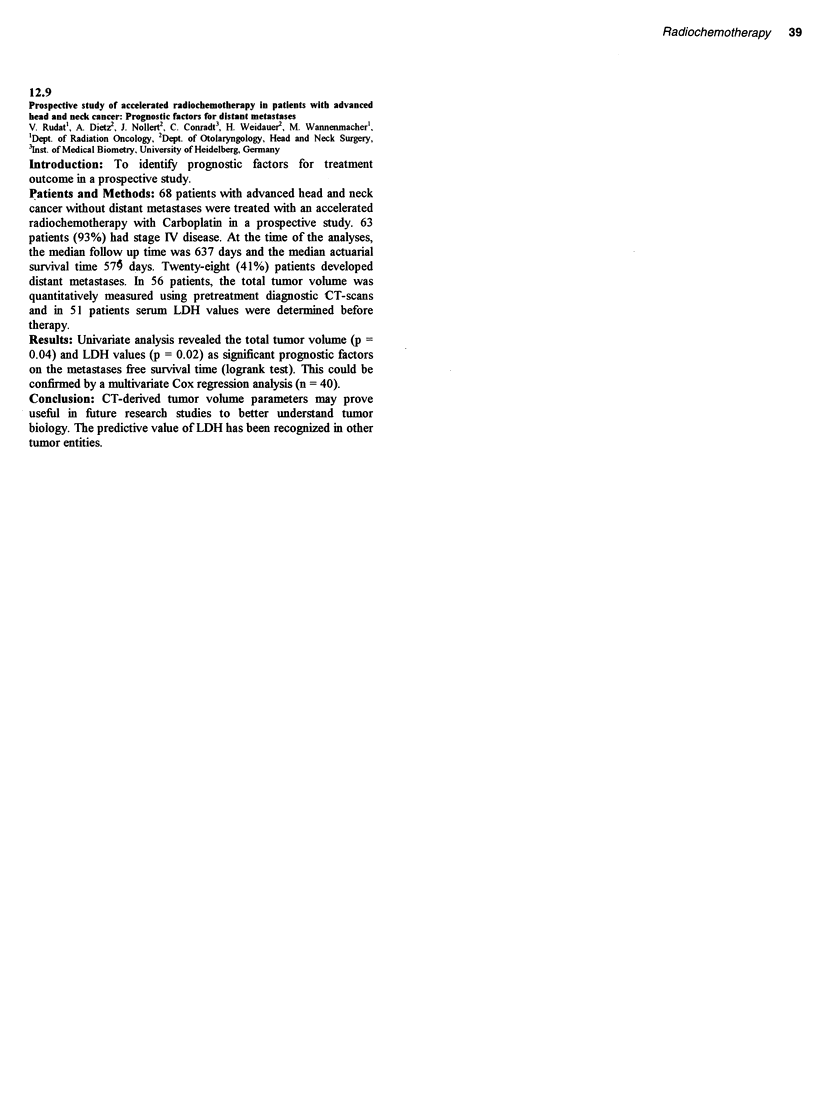# Radiochemotherapy

**Published:** 1998

**Authors:** 


**Outcome after comprehensive neck dissection with positive surgical margins**

L.E. Smeele, C.R. Leemans, R. Tiwari, I. van der Waal, G.B. Snow, Dept. Oral and Maxillofacial Surgery, Dept. ORL, University of Amsterdam,The Netherlands

**Introduction:** A positive surgical margin in a neck dissection specimen is a poor prognostic factor. Neck-recurrence rates may be improved with postoperative radiotherapy.

**Methods:** Out of a series of 1165 patients who underwent comprehensive neck dissections for squamous cell carcinoma of the lip/oral cavity, oropharynx, hypopharynx, or larynx between 1974-1995, 72 patients (6.1%) had positive margins at histopathological examination. During surgery, fixation of metastases to non-lymphatic structures was noted in 55 dissections. Adjuvant treatment consisted of radiotherapy with curative intent in 33, palliative radiotherapy in 13, and chemotherapy in 14 patients. Cumulative survival distributions were estimated by the Kaplan-Meier analysis and differences between groups were analysed with the Logrank test.

**Results:** Recurrence in the ipsilateral neck was 62.6% at five years and the median time to recurrence was 20 months. After treatment with radiotherapy with curative intent this was 44.2%. The remaining necks recurred in 84.2%. The difference between the two groups is statistically highly significant ($p<0.00001$).

**Conclusion:** Regional recurrence free rates after neck dissection with tumour at the margins are extremely poor, but can be improved considerably by a curative dose of radiotherapy postoperatively if this modality is still available.

### 12.2

**Is planned neck dissection indicated if chemotherapy is added to 60 Gy?**

G. Sanguineti[1], R. Corvo'[1], M. Benasso[2], A. Bacigalupo[1], G. Margarino[3], V. Vitale[1], Depts. of [1]Radiation Oncology, [2]Medical Oncology Ist, [3]Surgical Oncology, National Institute for Cancer Research, Genoa, Italy

**Introduction:** Planned neck dissection (PND) for large volume N2-3 neck disease is recommended after moderate dose radiotherapy (RT). The purpose of the present study is to assess if routine neck dissection is necessary even after chemotherapy alternated with 60 Gy.

**Methods:** From 1987 to 1995, 53 N1-3 heminecks from 43 patients with HN-SCC have been treated with alternated chemoradiotherapy (60 Gy at 2 Gy/fraction alternated with 4 cycles of cisplatin and 5-fluorouracil) at the National Institute for Cancer Research of Genoa. For living patients, median follow up is 37 months (range: 24-67.9 months).

**Results:** After chemoradiotherapy alone, 2-yr neck control probabilities (NCP) are 86+8%, 59+10% and 0 for N1, N2a-b and N3 neck stages, respectively ($p=0.005$). Two-year NCP for 33 complete responses is 85+8%, while, at the same time interval, it is 15+8% for 20 partial responses ($p<0.0001$). A further breakdown of NCP by neck stage and response shows no difference between N1 and N2a-b lesions in both partial ($p=0.48$) and complete responses ($p=0.54$). Five (9%) heminecks have developed severe (RTOG grade >2) subcutaneous late reactions.

**Conclusions:** PND is recommended even after moderate dose RT alternated with chemotherapy unless response is accounted for patients' selection.

### 12.3

**Neck lymph node biopsy did not increase the distant metastases in AJCC $T_{1-3}N_{1-3}$ nasopharyngeal carcinoma patients**

P.C. Pai[1], N.M. Tsang[1], C.F. Lin[2], C.K. Tseng[1], S. Tang[1], [1]Dept. of Rad. Oncology, [2]Dept. of Public Health, Chang Gung College of Medicine and Technology, Linkou, Taiwan

**Purpose:** To investigate whether the neck lymph node biopsy procedure increases the distant metastases rate in AJCC $T_{1-3}N_{1-3}$ nasopharyngeal carcinoma (NPC) patients. **Material and Methods:** From February 1979 to December 1991, 1181 NPC patients were treated in Chang Gung Memorial Hospital radiation oncology department. These patients completed the proposed course of nasopharyngeal treatment equal or greater than 64 Gy and were followed up for more than 5 years. Five hundred and forty-three patients belonged to AJCC $T_{1-3}N_{1-3}$ stage and in which 88 cases had neck lymph node operation procedure (aspiration 7, incision 39, excision 40, and dissection 2). **Results:** No statistical difference is recognized in age, operation method, or the time interval between operation and start of local treatment but sex, N stage, and neck area electron boost. **Discussion:** In this study, though it is suggested that neck lymph node operation before local-regional irradiation do not increases the distant metastatic rate in AJCC $T_{1-3}N_{1-3}$ NPC patients.

### 12.4

**Organ preservation in pharyngeal carcinoma presenting with advanced cervical metastasis**

A.S. Allal[1], P. Dulguerov[2], S. Bieri[1], W. Lehmann[2], J.M. Kurtz[1], [1]Dept. of Radiation Oncology, [2]Dept. of Head and Neck Surgery, University of Geneva, Switzerland

**Objective:** To evaluate treatment results in T1-T3 pharyngeal cancers presenting with extensive neck disease (N2-N3), using neck dissection followed by radical locoregional radiotherapy (RT). To compare these results with those obtained in comparable patients treated by radical RT alone.

**Patients and Intervention:** 41 patients with carcinomas of the oro- or hypopharynx, staged as T1-3 N2-3 M0 (AJCC stage IV), were treated with radical RT using a progressively accelerated concomitant boost schedule. RT was the sole treatment in 17 patients (group 2) and followed surgical neck dissection in 24 (group 1).

**Outcome:** Three-year locoregional control and overall survival. Post-operative complications, acute and late radiation effects.

**Results:** Actuarial locoregional control was 73% and 55% for groups 1 and 2, respectively ($p= 0.52$). After salvage surgery regional control was 78% and 69 % in groups 1 and 2, respectively ($p=0.8$), and the corresponding 3-year actuarial overall survival rates were 37% and 50% ($p= 0.42$). Severe postoperative complications were observed after neck dissection in 4 patients (16%). Acute toxicity during RT was similar in the two groups. Late toxicities were also similar, except for two patients in group 1 who developed severe laryngeal edema.

**Conclusion:** This atypical approach, consisting of neck dissection followed by radical RT to the primary tumor and neck, represents a valid treatment option in this subset of patients, allowing good control of advanced neck disease, while at the same time conserving pharyngolaryngeal function.

### 12.5

**Monitoring of 5-FU pharmacokinetics in cervical lymph node metastases from head and neck carcinomas by means of in vivo 19F MR spectroscopy**

H.P. Schlemmer[1], A. Dietz[2], M. Becker[1], P. Wollensack[2], P. Bachert[1], V. Rudat[3], B. Vanselow[2], M. Knopp[1], M. Wannenmacher[3], H. Weidauer[2], G. van Kaick[1], [1] German Cancer Research Center, [2,3]University of Heidelberg, Germany

**Introduction:** In patients with squamous cell carcinomas (SCC) of the. head and neck response rates may be improved by using combined radio/chemotherapy with 5-fluorouracil (5-FU). Purpose of this study is to evaluate the potential of in vivo $^{19}$F magnetic resonance spectroscopy ($^{19}$F MRS) for monitoring individual pharmacokinetics of 5-FU.

**Subjects and Methods:** Patients with advanced SCC of the oro- and hypopharynx ($T_3/T_4$; $N_2b,c/N_3$) were examined. All patients underwent an accelerated hyperfractionated concomitant-boost radio/chemotherapy. 5-FU (600 mg/[m$^2$*d]) and carboplatin (70 mg/[m$^2$*d]) were applied during week 1 and 5 of treatment. $^{19}$F MRS was performed with a 5 cm surface coil at 1.5 T on day 1 of the 1$^{st}$ and 2$^{nd}$ cycle of chemotherapy. $^{19}$F MR spectra were acquired during the 50 min infusion of 5-FU.

**Results:** In this ongoing study in 8/9 patients resonances of 5-FU, a-fluoro-b-alanine (FBAL) and 5-fluoro-5,6-dihydrouracil (DHFU) were detected. During the 2$^{nd}$ cycle of chemotherapy enhanced levels of intratumoral 5-FU were observed in 7/8 patients.

**Discussion:** Individual 5-FU pharmacokinetics can be monitored noninvasively using $^{19}$F MRS. The observed enhancement of 5-FU levels during the radio/chemotherapy indicate a modulation of 5-FU pharmacokinetics by irradiation.

### 12.6

**Rise of oxygenation in cervical lymph node metastasis of advanced cancer of the head and neck during the initial course of radiochemotherapy**

A. Dietz[1], V. Rudat[2], B. Vanselow[1], C. Conradt[3], M.J. Eble[2]; H. Weidauer[1], [1]Dept. of RL, [2]Dept. of Rad. Oncology, [3]Inst. of Med. Biometry, University of Heidelberg, Germany

**Introduction:** The therapeutic effect of radiotherapy depends to a significant extent on the degree of oxygenation of tumor tissue. It has been hypothesised that during radiation treatment, a reoxygenation of hypoxic tumor tissue takes place. To test this hypothesis, we have investigated wether reoxygenation in lymph node metastases could be determined by invasive partial oxygen pressure measurements.

**Patients and Methods:** Via a hyperdermic needle inserted transcutaneously into tumor positive lymph nodes, polarographic oxygen determinations (Eppendorf $pO_2$-histograph) were made on 18 patients with advanced squamous cell carcinomas of the oro- and hypopharyx. These measurements were performed before therapy and a week after onset of accelerated radio- or radiochemotherapy.

**Results:** Low $pO_2$ values before treatment (median 18.9 mmHg, average 18.6 mmHg) and a *hypoxic fraction* ($pO_2 < 5$ mmHg) of 40.3% (median) indicated manifest tumor hypoxia. After one week of treatment a significant increase in the median (25.5 mmHg) and the average $pO_2$ (24.1 mmHg) as well as a reduction in the hypoxic fraction (mean value of median shift: 13.4% $pO_2 < 5$ mmHg, $p < 0.03$) were observed.

**Conclusion:** Thus, invasive $pO_2$ histography fulfils the requirements for a method to confirm tumor hypoxia in head and neck tumors. The results obtained indicate that reoxygenation occurs during the initial phases of radio- and radiochemotherapy.

### 12.7

**Oxygen distribution in lymph node metastases, primary tumors and normal tissues in head and neck carcinomas**

M. Bloching[1], A. Becker[2], J. Dunst[2], A. Berghaus[1], [1]Dept. of ORL, [2]Dept. of Radio-Oncoclogy, University of Halle/Saale, Germany

**Background:** There is a correlation between the effect of radiation and the $pO_2$-tension in malignant tissues. In this study we determined the oxygen distribution in lmpyh node metastases (LN), primary tumors (PT) and the sternocleidomastoid muscle (SM) in patients with carcinomas of the head and neck region. We measured the $pO_2$-pressure at 19 patients with the Eppendorf $pO_2$-Histograph.

**Results:** The median $pO_2$ of primary tumors, neck node metastases as well as the sternocleidomastoid muscle showed a broad variance. The median $pO_2$-values in malignant tissues were in 14 of 15 cases below the value of SM. 80% of PT and more than 90% of LN showed a $pO_2$-pressure below 5 mm Hg. There was a strong correlation of the determined oxygenation of PT and LN.

**Conclusions:** The preliminary data of this cooperation-study showed a comparable oxygenation of advanced PT and their regional lymph node metastases in squamous cell carcinomas of head and neck region. This results may improve the knowledge about the relationsship of tumor oxygenation and malignant progression under radiation therapy.

### 12.8

**Taxotere© (Docetaxel) in the treatment of patients with locally advanced and metastatic head & neck cancer**

P. Schöffski, T. Weihkopf, A. Ganser, Dept. of Hematology/Oncology, Hannover University Medical School, Germany

**Background:** Taxotere© (Docetaxel) is a new cytotoxic agent obtained through a semisynthetic process from the needles of the European yew tree, *Taxus Baccata*. Taxotere© interferes with the rate and extent of microtubule assembly and disassembly as one major component of the cell proliferation process. The drug has been discovered in 1986, tested in clinical trials since 1990 and approved in 55 countries for treatment of solid tumors since 1995. Taxotere© is clinically active in a broad range of malignancies. **Safety and Toxicity:** Short-lasting neutropenia rarely complicated by fever or infections is the main dose limiting toxicity of the drug. Acute hypersensitivity reactions and fluid retention have been observed in early clinical trials, but are now markedly reduced by the use of corticosteroid co-medication. In contrast to other taxoids, Taxotere© has no clinically relevant cardiotoxicity. **Single-agent activity:** Taxotere© is commonly given at a dose of 100 mg/m$^2$ BSA as a one hour i.v. infusion every three weeks. The antitumor activity of single-agent Taxotere© in patients with head and neck cancer has been evaluated in a number of European, North American and Japanese trials, resulting in objective response rates of 23-42% in patients with metastatic disease or locoregional recurrences (Catimel et al. 1994, Fuji et al. 1995, Posner et al. 1996, Couteau et al. 1996, Ebihara et al. 1997). **Combination chemotherapy:** The combination of Taxotere© with other active antitumor agents appeared both feasible and clinically active in Phase I. Various regimens have been evaluated in recent Phase II trials in patients with advanced and/or metastatic head and neck cancer (Schöffski et al. 1996, Janinis et al. 1997, Posner et al. 1997). Objective responses of 55-100% have been reported in patients with locally advanced, recurrent or metastatic disease, including 25-67% complete remissions. **Future directions:** The good antitumor activity of Taxotere© combinations has led to the design of a number of randomized Phase III trials, which are currently initiated by cooperative study groups both in Europe and the U.S. These comparative studies will further define the role of this promising new agent for treatment of patients with head and neck cancer.

### 12.9

**Prospective study of accelerated radiochemotherapy in patients with advanced head and neck cancer: Prognostic factors for distant metastases**

V. Rudat[1], A. Dietz[2], J. Nollert[2], C. Conradt[3], H. Weidauer[2], M. Wannenmacher[1],
[1]Dept. of Radiation Oncology, [2]Dept. of Otolaryngology, Head and Neck Surgery, [3]Inst. of Medical Biometry, University of Heidelberg, Germany

**Introduction:** To identify prognostic factors for treatment outcome in a prospective study.

**Patients and Methods:** 68 patients with advanced head and neck cancer without distant metastases were treated with an accelerated radiochemotherapy with Carboplatin in a prospective study. 63 patients (93%) had stage IV disease. At the time of the analyses, the median follow up time was 637 days and the median actuarial survival time 579 days. Twenty-eight (41%) patients developed distant metastases. In 56 patients, the total tumor volume was quantitatively measured using pretreatment diagnostic CT-scans and in 51 patients serum LDH values were determined before therapy.

**Results:** Univariate analysis revealed the total tumor volume ($p = 0.04$) and LDH values ($p = 0.02$) as significant prognostic factors on the metastases free survival time (logrank test). This could be confirmed by a multivariate Cox regression analysis ($n = 40$).

**Conclusion:** CT-derived tumor volume parameters may prove useful in future research studies to better understand tumor biology. The predictive value of LDH has been recognized in other tumor entities.